# Immune profiles of pre-frail people living with HIV-1: a prospective longitudinal study

**DOI:** 10.1186/s12979-024-00416-5

**Published:** 2024-03-13

**Authors:** Lucy Kundura, Renaud Cezar, Sandrine Gimenez, Manuela Pastore, Christelle Reynes, Albert Sotto, Jacques Reynes, Clotilde Allavena, Laurence Meyer, Alain Makinson, Pierre Corbeau

**Affiliations:** 1 Institute of Human Genetics, CNRS-Montpellier University UMR9002, 141 rue de la Cardonille, Montpellier, 34396 France; 2 Immunology Department, Nîmes University Hospital, Place du Pr Debré, Nîmes, 30029 France; 3 Institute of Functional Genomics UMR5203 and BCM, CNRS-INSERM-Montpellier University, 141 rue de la Cardonille, Montpellier, 34396 France; 4 Infectious and Tropical Diseases Department, Nîmes University Hospital, Nîmes, France; 5 Montpellier University, Montpellier, France; 6 Infectious and Tropical Diseases Department, Montpellier University Hospital, Montpellier, France; 7 Service de Maladies Infectieuses, CHU de Nantes, Université de Nantes, Nantes, UE 1413 France; 8 INSERM CESP U1018, Le Kremlin Bicêtre, France; 9 Department of Public Health and Epidemiology, Bicêtre Hospital, AP-HP, Paris-Saclay University, Le Kremlin-Bicêtre, France

## Abstract

**Background:**

People living with HIV (PLWH) are at risk of frailty, which is predictive for death. As an overactivity of the immune system is thought to fuel frailty, we characterized the immune activation profiles linked to frailty.

**Methods:**

We quantified twenty-seven activation markers in forty-six virological responders (four females and forty-two males; median age, 74 years; median duration of infection, 24 years; median duration of undetectability, 13 years), whose frailty was determined according to the Fried criteria. T cell and NK cell activation was evaluated by flow cytometry, using a panel of cell surface markers. Soluble markers of inflammation, and monocyte activation and endothelial activation were measured by ELISA. The participants’ immune activation was profiled by an unsupervised double hierarchical clustering analysis. We used ANOVA p-values to rank immunomarkers most related to Fried score. A Linear Discriminant Analysis (LDA) was performed to link immune activation markers to frailty.

**Results:**

41% of the participants were pre-frail, including 24% with a Fried score of 1, and 17% with a Fried score of 2. ANOVA identified the 14 markers of T cell, monocyte, NK cell, endothelial activation, and inflammation the most linked to Fried 3 classes. The LDA performed with these 14 markers was capable of discriminating volunteers according to their Fried score. Two out of the 5 immune activation profiles revealed by the hierarchical clustering were linked to and predictive of pre-frailty. These two profiles were characterized by a low percentage of CD4 T cells and a high percentage of CD8 T cells, activated CD4 T cells, CD8 T cells, and NK cells, and inflammation.

**Conclusions:**

We identified a particular immune activation profile associated with pre-frailty in PLWH. Profiling participants at risk of developing frailty might help to tailor the screening and prevention of medical complications fueled by loss of robustness. Further studies will indicate whether this frailty signature is specific or not of HIV infection, and whether it also precedes frailty in the general population.

**Supplementary Information:**

The online version contains supplementary material available at 10.1186/s12979-024-00416-5.

## Introduction

Frailty is the consequence of a decline in physiologic reserve resulting in vulnerability to stressors [1]. The frequency of this geriatric syndrome remains higher in virologically suppressed people living with HIV (PLWH) than in HIV-uninfected counterparts [2]. As frailty is predictive of falls, hospitalization and death in PLWH [3, 4], it is of prime importance to better understand its pathophysiology.

Frailty is linked to age [5, 6], smoking [7], low socioeconomic status, and multimorbidity, though inconstantly [8–10]. It is more common in PLWH who present low CD4 nadir [9–12], a low CD4 count [7, 11], high CD8 count [7], low CD4/CD8 ratio [7, 11], high frequencies of activated CD4 T cells and CD8 T cells [7], and markers of monocyte activation like soluble CD14 [7] and soluble CD163 [12]. A poor CD4 count has even been shown to predict pre-frailty [11].

Frailty is also associated with comorbidities, including low bone density [13], neurocognitive impairment [14], depression [9], diabetes [6], kidney disease [6], hepatitis C virus [15], and cytomegalovirus [16] infections.

Immune activation, which is fueled by aging [17], low socioeconomic status [18], morbidity [19], and HIV infection [20] is thought to fuel frailty. Accordingly, markers of inflammation, such as for instance C-reactive protein [21], TNFα [21], soluble TNF receptors [21, 22], IL-6 [7, 22–25] and IFNγ [25] are linked to frailty in PLWH.

Here, we measured twenty-seven activation markers in forty-six virologic responders aged over 70 whose frailty had been determined, and identified the immune signatures related to pre-frailty using various biostatistical approaches.

## Materials and methods

### Study design

This was a substudy of the ANRS EP66 SEPTAVIH study [5]. People living with HIV-1, aged over 70 years, and treated for HIV-1 infection for at least 12 months were recruited at the University Hospitals of Montpellier and Nîmes. An Ethics Committee had approved this study and all patients had provided written informed consent (ID-RCB: 2018-A03100-55). The trial was registered on ClinicalTrials.gov (NCT03958786).

### Bioclinical evaluation

Frailty was assessed at Month 0 and Month 12 with Fried Frailty Phenotype that measures 5 clinical parameters (weight loss, exhaustion, low physical activity, slow gait and weakness of hand grip [1]) unintentional weight loss, grip strength [26], exhaustion (evaluated by questions n°7 and n°20 on the Center for Epidemiologic Studies Depression scale), walking speed (4-meter gait speed test, adjusted for gender and height), and physical activity using the International Physical Activity Questionnaire [27]. Participants were classified as robust (no criteria), pre-frail (1 or 2 criteria), and frail (3 or more criteria). The Veterans Aging Cohort Score (VACS) index, a morbi-mortality score, was calculated according to age, CD4 count, plasma HIV-1 RNA level, hemoglobin, liver fibrosis (FIB-4 index), estimated Glomerular Filtration Rate, and the presence or absence of hepatitis C infection. The number of morbidities - including high blood pressure, cardiovascular events, diabetes mellitus, dyslipidemia, chronic kidney disease, cancer and chronic respiratory disease - was quantified. The socioeconomic status was determined by the Assessment of Precariousness and Health Inequalities in Health Examination Centers (EPICES) index [28].

### Flow cytometry

Monoclonal antibodies conjugated with fluorescein isothiocyanate (FITC), phycoerythrin (PE), energy-coupled dye (ECD), PE-Cyanine5.5 (PC5.5), PE-Cyanine7 (PC7), Alexa Fluor 647 (AF647), allophycocyanine (APC), APC/Alexa700, or APC/Alexa750 were purchased from Beckman Coulter (Supplementary Table 1). Antibodies were used in the following combinations; CD57-FITC/CD279-PE/CD45RA-ECD/CD28-PC5.5/CD27-PC7/CD8-APC/CD4-APC700/CD3-APC750 to identify naïve (CD45RA + CD27+), central memory (CD45RA-CD27+), effector memory (CD45RA-CD27-), senescent (CD57 + eventually devoid of CD28 and CD27) and exhausted (CD279+) CD4 and CD8 T cells, CD8-APC/CD4-APC700/CD3-APC750/CD38-PE/HLADR-PC7 to identify activated (HLA-DR + and/or CD38+) CD4 and CD8 T cells, CD3-APC750/CD16 APC/HLADR-PC7/CD56-PC5.5/CD57-FITC to identify activated (HLA-DR+), senescent (CD57+), and CD56- NK cells. Whole blood collected in ethylenediaminetetraacetic acid tubes was stained within one hour for 10 min at room temperature in the dark with a cocktail of antibodies and fixed using an IMMUNOPREP reagent system kit system and TQ Prep automate (Beckman Coulter). For FoxP3 intracellular labelling, cells were permeabilized and fixed with PerFix-nc kit (Beckman Coulter) according to the manufacturer’s guidelines. A minimum of 20,000 cells were run on a Navios flow cytometer and results were analyzed using Kaluza software (Beckman Coulter).

### Soluble markers in plasma

Soluble TNF receptor I (sTNFRI), soluble CD163 (sCD163) (Quantikine, R&D systems), tissue Plasminogen Activator (tPA), and soluble Endothelial Protein C Receptor (sEPCR) (Asserachrom, Stago, USA) were quantified by ELISA.

### Statistical analysis

GraphPad Prism 10 software was used to perform the statistical analyses and graph representation. Data were tested for normal distribution using the D’Agostino and Pearson test. A Chi-square test was used to compare pre-frailty frequency and incidence between volunteers according to their immune activation profiles. ANOVA or the Kruskal Wallis test, as appropriate, was applied to evaluate markers differences between groups. As previously described [29], an unsupervised double hierarchical clustering analysis of patients and markers was carried out for patients, using the Euclidian distance to measure the distance between individuals, and another one for markers, using 1-abs (correlation) as a distance. For both of them, Ward’s minimum variance method was used as a means of linkage. We then generated a heatmap using the classification of patients and markers. For the supervised analysis, a linear discriminant analysis was applied to each solution (immunological marker combinations) using the R package MASS [30]. This supervised method creates linear combinations of all variables in order to optimize the separation of known classes. Novel features were obtained by maximizing between-class variability (separating classes as far as possible) and minimizing the within-class variability (so that classes were as close as possible) to assign observations to target classes (prediction). The number of new discriminant axes is equal to the number of target classes minus one. Cross-validation was performed by separating the observations into two groups (a training dataset on which the model is optimized and a test dataset on which the model is validated) for greater robustness of results. Logistic regression was applied to assess association between each immunomarker and each cofactor to predict frailty status.

## Results

### Study subjects

Forty-six people living with HIV-1 were recruited. Their bioclinical characteristics are reported in Table 1. Twenty-seven (59%) of them were robust (Fried score = 0), nineteen (41%) were pre-frail, and none were frail. Among these pre-frail patients, eleven (24%) had one Fried criterion, and 8 (17%) had two Fried criteria. Pre-frail participants tended to be older than robust participants (Table 1). Their EPICES and VACS index scores were non-significantly higher and their CD4:CD8 ratio non-significantly lower than those of the other participants (Table 1).

**Table 1:** Characteristics of study participants. Variables with normal distribution are described by their mean ± standard deviation (SD) and the differences calculated using an unpaired t test. Variables with non-normal distribution are described by their median and interquartile range (IQR) and the differences calculated using a Mann-Whitney test

	All	Robust	Pre-frail	Difference between pre-frail and non-pre-frail (p)
Sample size, n (%)	46	27 (59%)	19 (41%)	
Age, years (mean *±* SD)	74.4 *±* 3.2	73.6 *±* 2.6	75.5 *±* 3.9	0.058
Female (%)	4	2 (50%)	2 (50%)	0.712
Waist:Hip ratio (mean *±* SD)	1.00 ± 0.08	0.98 ± 0.06	1.03 ± 0.10	0.299
Current CD4 count, cells/µL (mean *±* SD)	592 *±* 245	622 ± 242	550 ± 248	0.335
Current CD4/CD8 ratio (median [IQR])	0.97 [0.64–1.36]	1.01 [0.76–1.44]	0.77 [0.54–1.06]	0.088
Pretherapeutic CD4 count, cells/µL (mean *±* SD)	278 *±* 171	255 ± 141	308 ± 206	0.353
Duration of infection, years (median [IQR])	24.5 [19.1–26.8]	23.8 [13.7–26.7]	25.0 [23.6–27.2]	0.778
Duration of undetectability, years (mean *±* SD)	8.9 ± 7.2	9.3 ± 7.2	8.5 ± 7.4	0.766
Number of comorbidities (median [IQR])	4.0 [3.0–5.0]	4.0 [2.7-5.0]	4.0 [3.0–6.0]	0.288
EPICES score (mean *±* SD)	22.8 ± 14.8	19.4 ± 12.9	27.8 ± 16.3	0.071
VACS index score (median [IQR])	39.0 [33.0-47.5]	38.0 [33.0–43.0]	39.0 [33.0–52.0]	0.105

We measured the percentages of CD4 T cells and CD8 T cells, activated (CD38 and/or HLA-DR+), exhausted (PD-1+), senescent (CD57+, eventually CD27-, and eventually CD28-), naïve (CD45RA + CD27+), central (CD45RA-CD27+) and effector (CD45RA-CD27-) memory CD4 T cells and CD8 T cells, of NK cells, activated (HLA-DR+), dysfunctional (CD56-), and senescent (CD57+) NK cells (Supplementary Figs. 1 and 2). We then quantified in plasma sCD163 (monocyte activation), sTNFRI (inflammation), tPA, and sEPCR (endothelium activation).

We used two different approaches to characterize the immune profile of pre-frail patients, one supervised and one non-supervised.

### Supervised immune profiling of patients

**Fig. 1:**
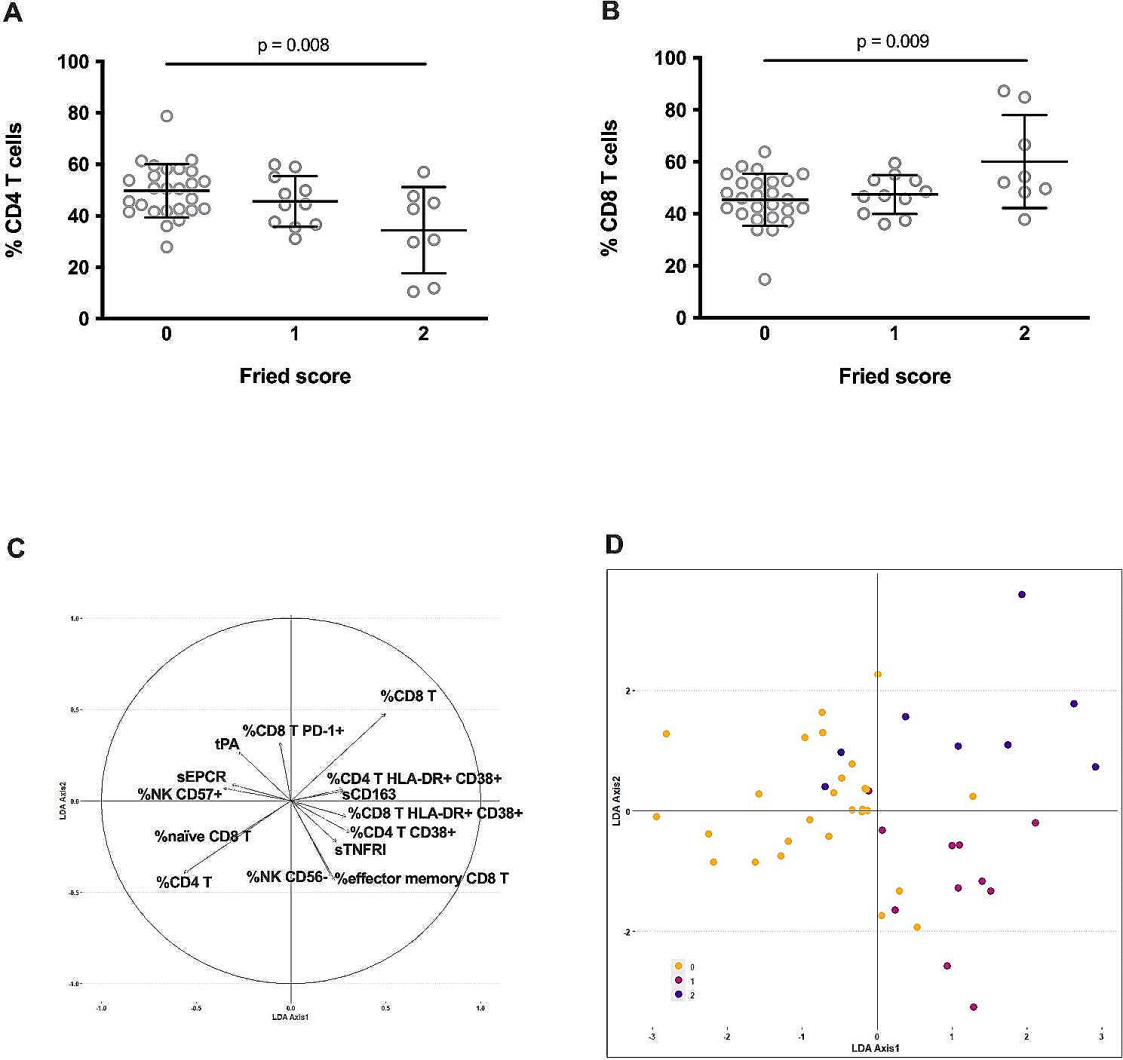
Frequencies of CD4 T cells (**A**) and CD8 T cells (**B**) cells in participants with Fried score of 0, 1, and 2. ANOVA was used to compare differences. Immune activation marker map resulting from the bidimensional representation of the samples into the new subspace generated by the first two LDA axes (**C**). LDA axis 1 globally separates robust samples from frailty ones, whereas LDA axis 2 allows splitting of the two levels of frailty, demonstrating a clear association between immune activation markers and Fried scores (0, yellow circle; 1, red circle, 2, purple circle, (**D**). Correlation of each feature to the two LDA axes shows markers mostly associated to the discrimination of the groups

We used a supervised approach to directly characterize the immune activation markers linked to pre-frailty. Looking for differences in the various activation markers according to pre-frailty, we observed a global difference in the frequencies of CD4 T cells (*p* = 0.008, Fig. 1A; Table 2) and CD8 T cells (*p* = 0.009, Fig. 1B; Table 2) between participants with a Fried score of 0, 1 or 2. Yet, these differences were not significant following multiple test correction. We also observed differences in 12 other markers, i.e., the frequencies of HLA-DR + CD4 T cells, HLA-DR + CD38 + CD4 T cells, naïve CD8 T cells, effector memory CD8 T cells, HLA-DR + CD38 + CD8 T cells, PD-1+ CD8 T cells, CD56- NK cells, CD57 + NK cells, and the plasma levels of sTNFRI, sCD163, sEPCR, and tPA (Table 2). Yet, these differences did not either reach statistical signifance. We performed a linear discriminant analysis (LDA) using these 14 markers more related to Fried score in 3 classes (Fried score O, 1 or 2) (Fig. 1C). This analysis enabled us to discriminate robust participants from pre-frail participants (Fig. 1D, axis 1), and participants with a Fried score of 1 and 2 (Fig. 1D, axis 2). We set logistic regressions to predict frailty status with each of the 14 selected markers and adjusted for three cofactors, age, socioeconomic status and the number of comorbidities. None of the interactions between immunomarkers and these cofactors was significant, suggesting that association of the Fried score with the immunomarkers did not depend on the level of any of the three cofactors. We concluded that the association between each immunomarker and pre-frailty was independent of age, socioeconomic status, or the number of comorbidities.

**Table 2:** Differences in various activation markers according to pre-frailty. SD, standard deviation; IQR, interquartile range. Differences were evaluated using ANOVA

	Fried score			Difference between the 3 groups (p)
	0	1	2	
% CD4 T cells (mean *±* SD)	50.1 ± 10.4	45.6 ± 9.8	34.4 ± 16.8	0.008
% CD8 T cells (mean *±* SD)	45.2 ± 9.9	47.5 ± 7.4	60.1 ± 17.9	0.009
% NK cells CD56- (mean *±* SD)	15.5 ± 11.0	25.4 ± 21.4	13.7 ± 5.3	0.106
% effector memory CD8 T cells (mean *±* SD)	9.5 ± 7.0	14.9 ± 10.9	8.4 ± 6.1	0.126
tPA, ng/mL (mean *±* SD)	14.7 ± 6.7	11.2 ± 3.3	14.0 ± 4.7	0.223
% naïve CD8 T cells (median [IQR])	29.7 [19.8–42.2]	23.2 [19.0-35.2]	22.6 [21.6–25.6]	0.233
% NK cells CD57+ (mean *±* SD)	52.4 ± 18.1	41.8 ± 22.2	43.4 ± 24.3	0.234
% CD4 T cells CD38+ (mean *±* SD)	54.1 ± 10.8	60.7 ± 14.2	57.3 ± 10.7	0.282
sEPCR µg/mL (median [IQR])	117.2 [75.9-170.5]	103.1 [81.5-137.1]	95.4 [83.6-128.9]	0.338
sTNFRI, ng/mL (median [IQR])	1.30 [1.20–1.50]	1.60 [1.20–1.90]	1.35 [1.20–1.57]	0.368
% CD8 T cells HLA-DR + CD38+ (mean *±* SD)	35.0 ± 16.2	42.1 ± 9.9	40.2 ± 20.0	0.375
% CD8 T cells PD-1+ (mean *±* SD)	47.5 ± 23.2	38.7 ± 22.8	53.3 ± 24.4	0.394
% CD4 T cells HLA-DR + CD38+ (mean *±* SD)	7.6 ± 4.8	9.2 ± 6.6	10.2 ± 6.3	0.424
sCD163, pg/mL (mean *±* SD)	576.0 ± 213.5	668.5 ± 318.8	714.3 ± 496.7	0.465

### Non-supervised immune profiling of patients

**Fig. 2:**
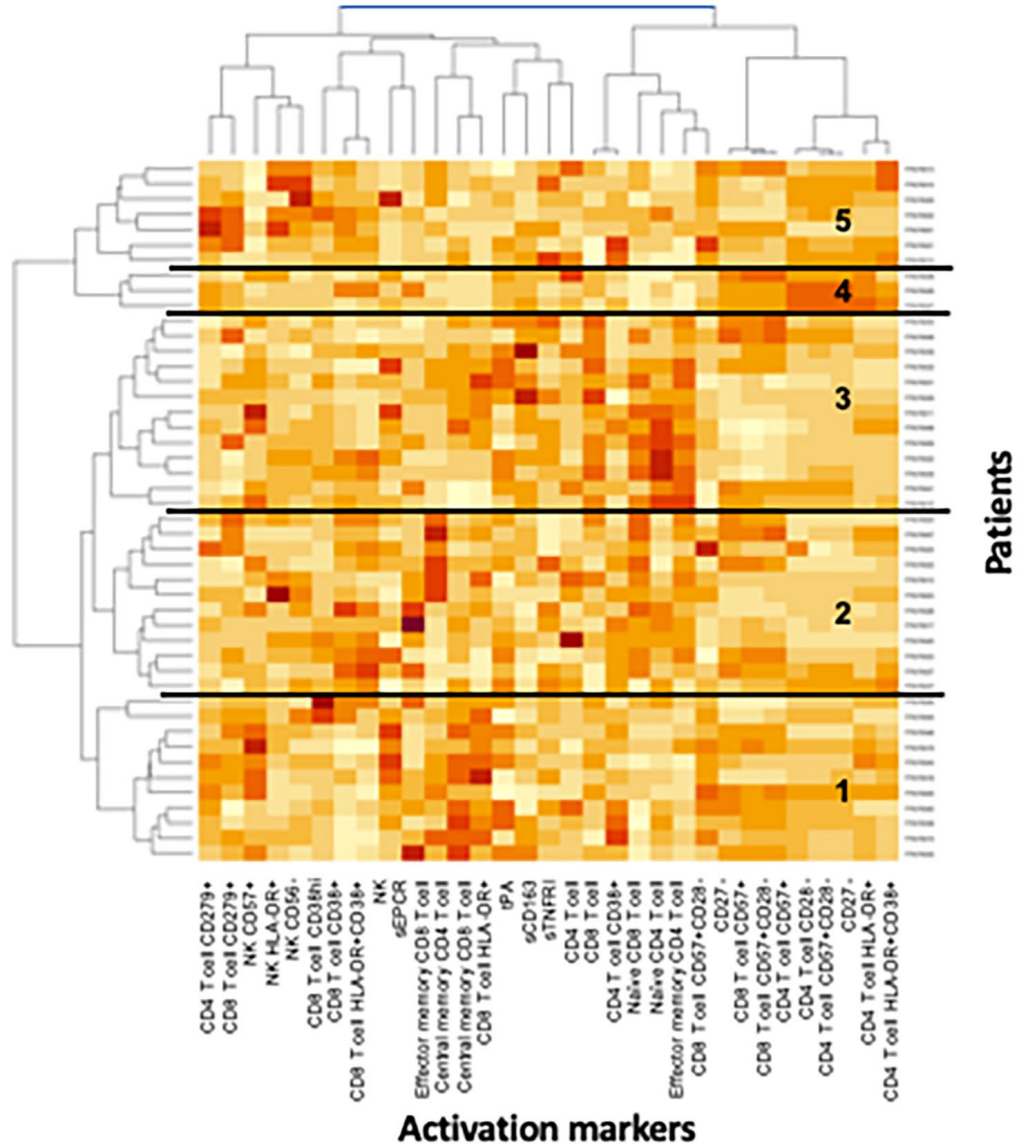
Heatmap showing the hierarchical clustering of activation markers (vertical) and participants (horizontal) according to their activation profile. Each profile number is indicated

In order to confirm the results of the supervised immune profiling, and to look for links not unveiled by this approach, we then performed a non-supervised immune profiling. This method allows the clustering of parameters according to their similarities and differences without any previous assumption about the role of a single biomarker or a specific group of biomarkers. We sorted the patients and markers via two independent hierarchical clustering analyses. Five different immune activation profiles were thus identified (Fig. 2).

**Fig. 3:**
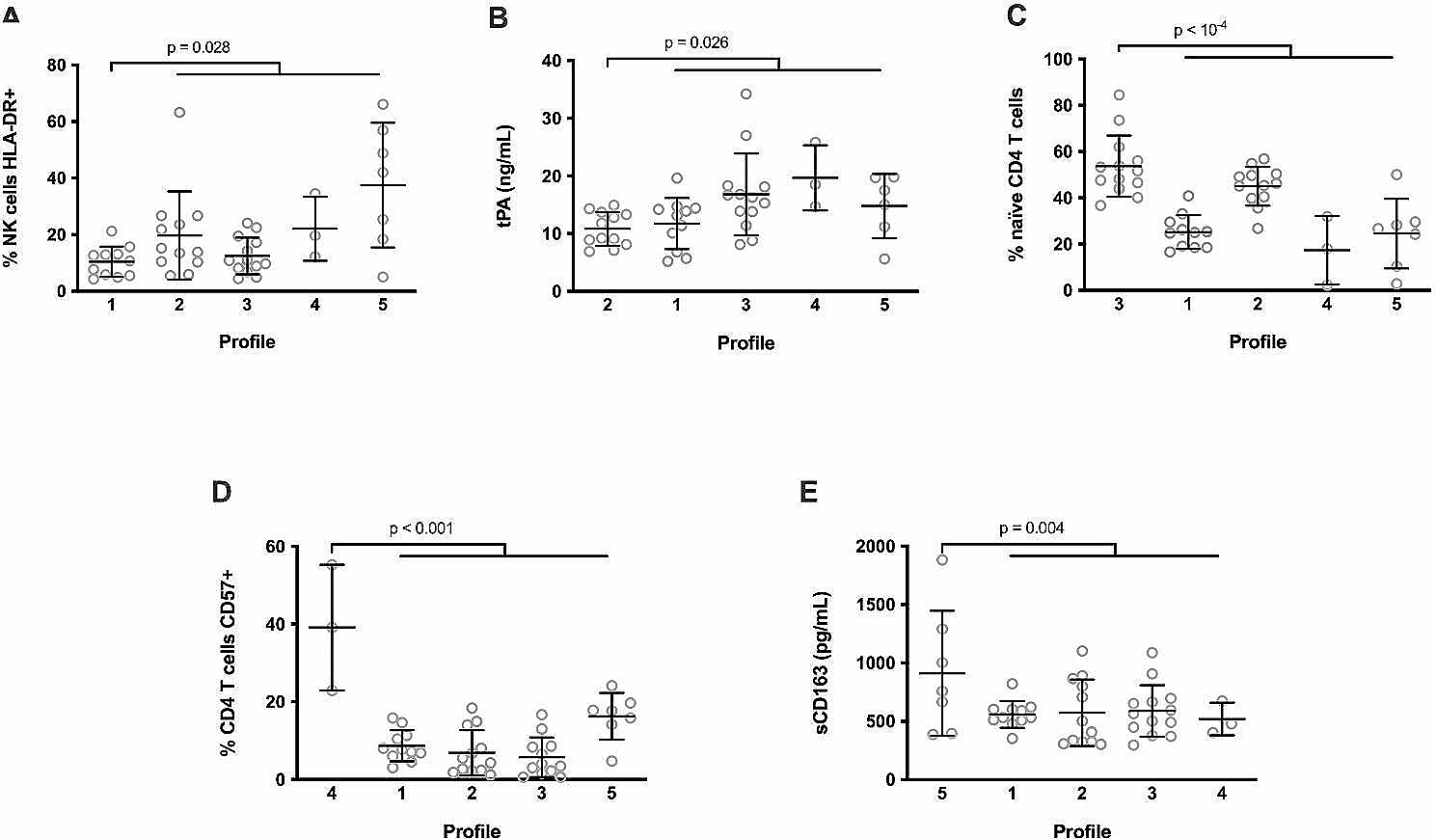
Characterization of the five immune activation profiles. Difference in the frequency of activated NK cells between Profile 1 participants and the other participants (**A**). Difference in tPA plasma level between Profile 2 participants and the other participants (**B**). Difference in the frequency of naïve CD4 T cells between Profile 3 participants and the other participants (**C**). Difference in the frequency of CD57-expressing CD4 T cells between Profile 4 participants and the other participants (**D**). Difference in sCD163 plasma level between Profile 5 participants and the other participants (**E**). Differences were evaluated using an unpaired t test or a Mann-Whitney test, as appropriate

We then looked for activation markers able to characterize each profile. Profile 1 participants which presented the lowest frequency of activated (HLA-DR+) NK cells (median: 10.7, interquartile range (IQR): 5.5–13.1 versus 15.2, 9.7–25.4%, *p* = 0.028, Fig. 3A). Profile 2 was distinguishable by a low level of tPA (mean ± standard deviation (SD): 10.8 ± 2.9 versus 14.8 ± 6.3 ng/mL, *p* = 0.026, Fig. 3B). Participants with Profiles 3 and 4 had the highest percentages of naïve CD4 T cells (mean ± SD: 53.7 ± 13.2 versus 31.6 ± 14.4%, *p* < 10^− 4^, Fig. 3C) and CD57 + CD4 T cells (mean, IQR: 39.1, 22.9–55.3 versus 6.8, 3.0-14.1%, *p* < 0.001, Fig. 3D), respectively. Finally, a high plasma concentration of the monocyte activation marker sCD163 characterized Profile 5 (mean ± SD: 911.7 ± 209.2 versus 570.2 ± 535.6 ng/mL, *p* = 0.004, Fig. 3E).

### Identification of immune activation profiles linked to pre-frailty as determined by unsupervised clustering

**Fig. 4:**
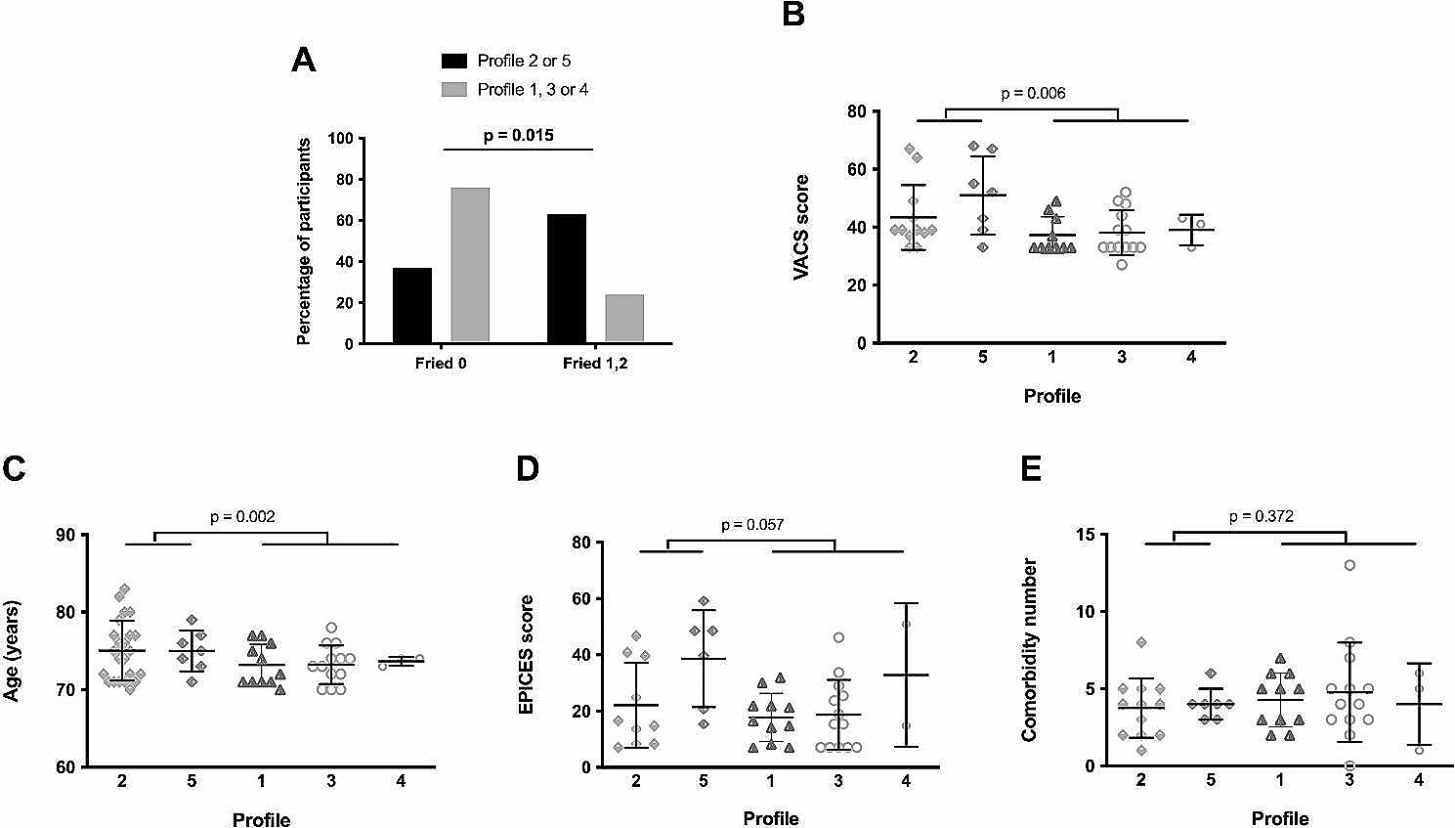
Proportion of robust and pre-frail participants in Profiles 2 and 5 (closed histogram) and the other Profiles (shaded histogram). The difference was evaluated using a Chi-square test (**A**). VACS score (**B**), age (**C**), EPICES score (**D**), and comorbidity frequency (**E**) in HIV participants with different immune activation profiles. Differences were evaluated using an unpaired t test or a Mann-Whitney test, as appropriate

Thereafter, we wondered whether any of the immune activation profile(s) we had unveiled was (were) linked to pre-frailty. Indeed, we observed that Profile 2 and 5 patients were more often pre-frail than the other patients (*p* = 0.015, Fig. 4A). Moreover, these patients presented higher VACS scores (*p* = 0.006, Fig. 4B), were older (*p* = 0.002, Fig. 4C), and tended to have a lower socioeconomic status (*p* = 0.057, Fig. 4D) than patients with Profiles 1, 3 or 4. Yet, they did not present more comorbidities than the other patients (*p* = 0.372, Fig. 4E).

**Fig. 5:**
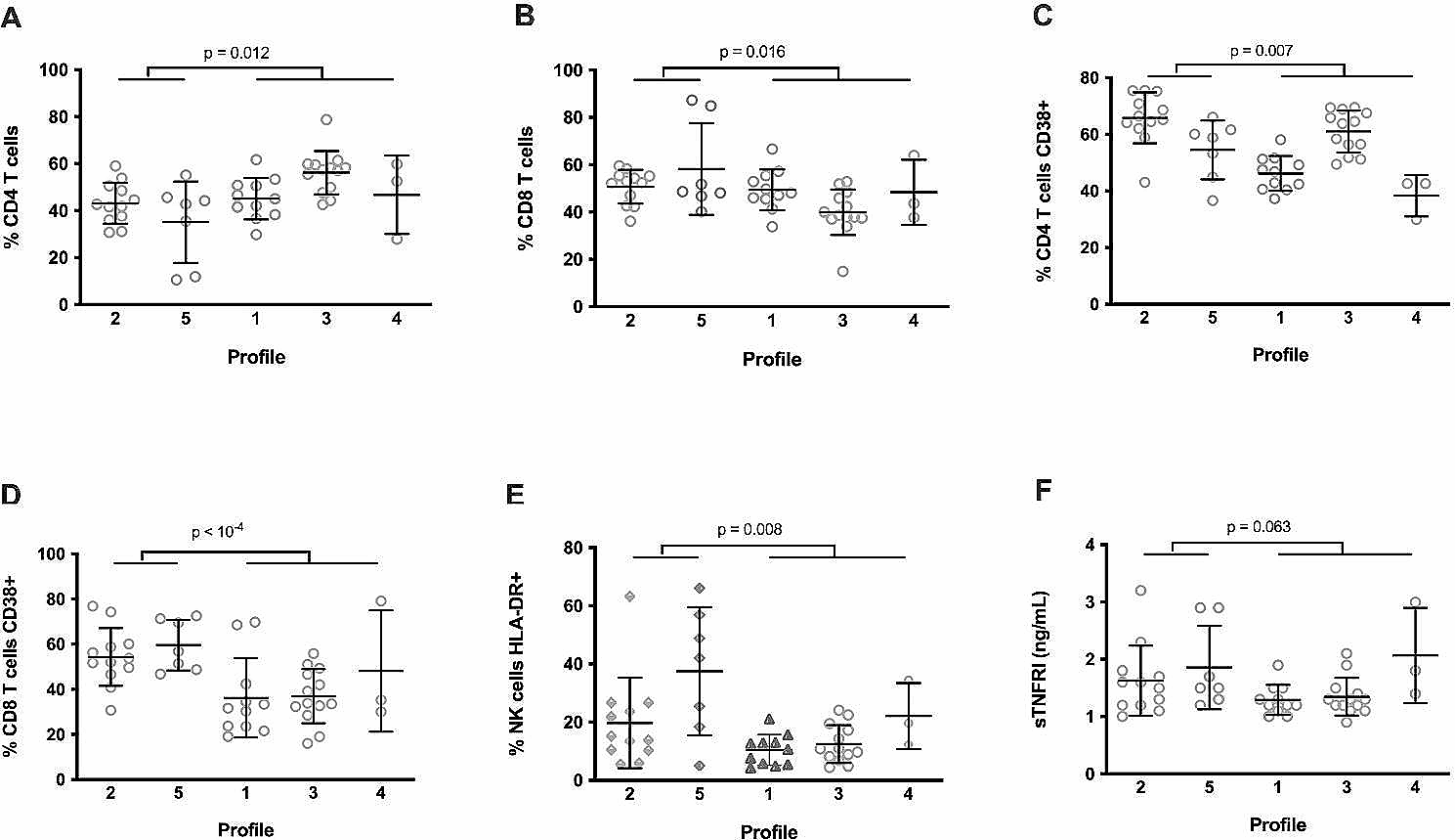
Characterization of the immune activation Profiles 2 and 5. Differences in the frequency of CD4 T cells (**A**), CD8 T cells (**B**), CD38-positive CD4 T cells (**C**), CD38-positive CD8 T cells (**D**), activated NK cells (**E**), and in sTNFRI plasma level (**F**) between Profiles 2 and 5 and the other profiles. Differences were evaluated using an unpaired t test or a Mann-Whitney test, as appropriate

**Fig. 6:**
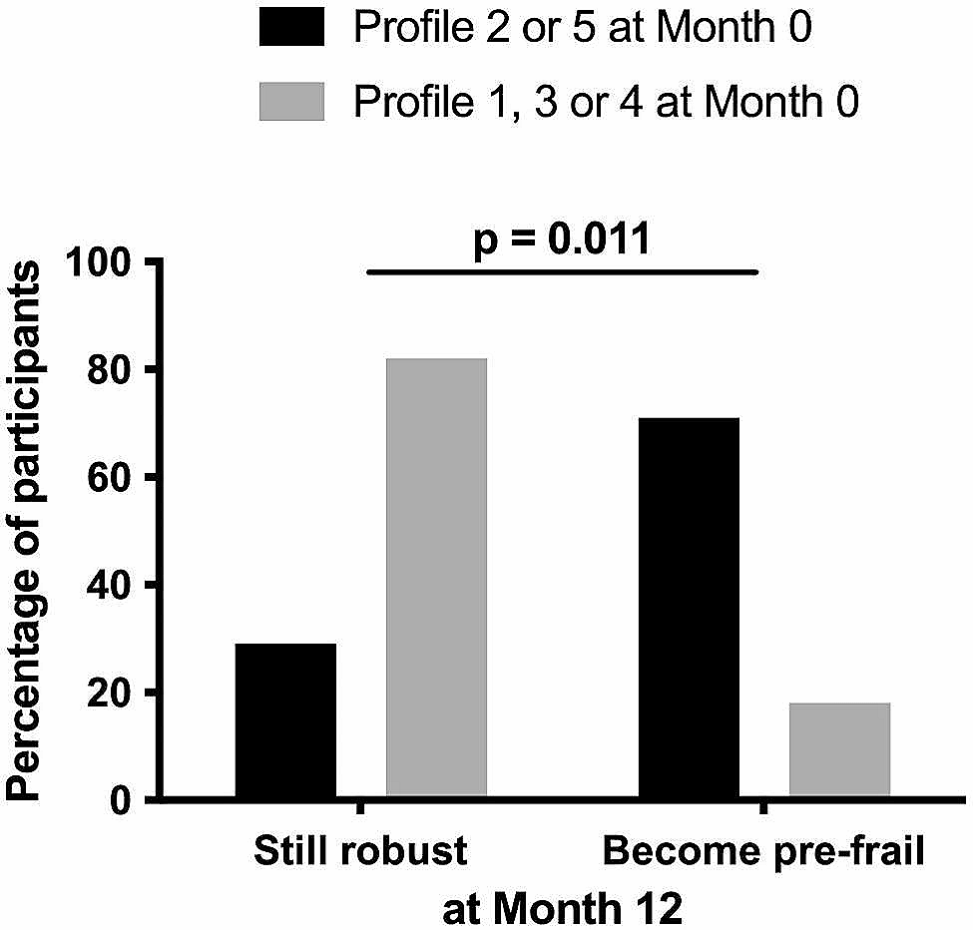
Proportion of participants robust at Month 0 becoming pre-frail or not over one year according to their initial immune activation profiles (Profiles 2 and 5, closed histogram, Profiles 1, 3, and 4, shaded histogram). The difference was evaluated using a Chi-square test

Next, we further characterized immune Profiles 2 and 5. Compared with the other volunteers, Profiles 2 and 5 patients presented a low percentage of CD4 T cells (Fig. 5A) and a high percentage of CD8 T cells (Fig. 5B). Their frequencies of activated (CD38+) CD4 T cells (Fig. 5C) and CD8 T cells (Fig. 5D) were high, as well as their frequency of activated (HLA-DR+) NK cells (Fig. 5E). They also presented a marker of inflammation (high sTNFRI, Fig. 5F). Finally, we wondered whether Profiles 2 and 5, as well as being linked to prefrailty, might also predict it. To answer this question, we evaluated frailty in the forty-six participants again, 12 months later. Strikingly, the pre-frailty incidence was higher over a year in robust patients with Profiles 2 and 5 than in robust patients with other profiles (71% vs. 18%, *p* = 0.011). Out of seven Profile 2 and 5 patients with a Fried score of 0 at Month 0, four reached a score of 1 and one a score of 2, whereas out of 17 Profile 1, 3, and 4 patients with a Fried score of 0 at Month 0, only three reached a score of 1 (Fig. 6).

## Discussion

Here, using two different approaches, one supervised and the other non-supervised, we identified the same set of immune activation markers linked to pre-frailty in efficiently treated PLWH. Indeed, on the one hand, the two immune activation Profiles (2 and 5) associated with pre-frailty presented a low proportion of CD4 T cells, a high proportion of CD8 T cells, markers of CD4 T cell (CD38+), CD8 T cell (CD38+), NK cell (HLA-DR+), and overproduction of the main inflammatory cytokine TNFα (sTNFRI). On the other hand, the scarcity of CD4 T cells, abundance of CD8 T cells, CD4 T cell activation (HLA-DR+, HLA-DR + CD38+), activation (HLA-DR + CD38 + CD8 T cells), differentiation (low percentage of naïve and high percentage of effector memory CD8 T cells), exhaustion (PD-1 + CD8 T cells) of CD8 T cells, NK maturity/senescence (CD57+), monocyte activation (sCD163) as well as inflammation (sTNFRI) were directly linked to the Fried score.

Thus, both analyses pointed to a low frequency of CD4 T cells, a high frequency of CD8 T cells, CD4 T cell, CD8 T cell, NK cell activation, as well as inflammation in pre-frail patients. As tPA was increased in Profile 2, and sEPCR higher in pre-frail than in robust volunteers, there was also a tendency towards endothelial activation.

In addition to this concordance between markers associated with pre-frailty obtained via two independent strategies, our results were in line with data from the literature. As a matter of fact, low CD4 counts [7, 11], high CD8 counts [7], high frequencies of activated CD4 T cell and CD8 T cells [7], high levels of soluble CD163 [12], and markers of inflammation (C-reactive protein [21], TNFα [21], soluble TNF receptors [21, 22], IL-6 [7, 22–25], IFNγ [25]) have been reported in frail PLWH.

These correlations are not specific to PLWH. Indeed, in the general population, inflammation is linked to frailty [31] and is predictive of neurocognitive impairment and survival [32, 33]. Even reduced CD4 T cell counts and increased CD8 T cell counts have been noticed in the overall population of frail people [34], in addition to T cell differentiation [35] which also predicts frailty [36] along with T cell senescence [37]. Concerning monocytes, neopterin, a marker of monocyte and macrophage activation, had been associated with a decrease in robustness in the population as a whole [38].

One of the limitations of the present study is that it is observational. Therefore, it is impossible to draw definitive conclusions on causative links between immune activation and frailty. Indeed, aging may favor immune activation and frailty independently. However, there are many reasons to think that inflammation might fuel frailty. First, inflammation paves the way for sarcopenia [39], a component of frailty [40], particularly via the downregulation of the expression and function of insulin-like growth factor I, and thereby of muscle maintenance and regeneration [41]. Second, inflammatory cytokines induce a decrease in neurogenesis [42] which may exacerbate frailty. And third, chronic inflammation, by interfering with iron and erythropoietin metabolisms, causes anemia which may participate in frailty [43]. As for chronic T cell activation, and its consequence, T cell differentiation, they generate T cell senescence, and senescent T cells produce soluble factors aggravating inflammation [44]. Activated monocytes and macrophages may also worsen inflammation by producing proinflammatory cytokines, and decrease the production of neurotransmitters involved in motor regulation by producing neopterin and kynurenines [35].

Finally, an additional argument for a role of immune activation in the development of frailty is the observation that in our study Profiles 2 and 5 were associated with pre-frailty occurrence 12 months later.

Our work unveils an immune activation signature linked to HIV-associated pre-frailty. It would now be of interest to perform the same kind of study in a non-specific population to test whether a similar signature is generally present in frailty, as we have shown that this is the case with insulin resistance [30, 45]. In addition to its pathophysiological interest, the present study may have an implication for the management of frailty. As the frail phenotype is at least partly reversible [46], it is important to identify predictive markers. Immune activation Profiles 2 and 5 are clear candidates.

### Electronic supplementary material

Below is the link to the electronic supplementary material.

Supplementary Material 1

## Data Availability

No datasets were generated or analysed during the current study.
