# Supplementary material for: Immune profiles of pre-frail people living with HIV-1: a prospective longitudinal study
Source: Immun Ageing. 2024 Mar 13;21:20. doi: 10.1186/s12979-024-00416-5 (PMC10935995; doi:10.1186/s12979-024-00416-5)
Supplement: Supplementary file 1 — Supplementary Material 1 [file 12979_2024_416_MOESM1_ESM.docx]

**Supplementary file**

|  |  |  |  |  |  |
| --- | --- | --- | --- | --- | --- |
|  | Receptor/Specificity | Fluorochrome | Clones | Manufacturier |  |
|  | CD57 | Fluorescein Isothiocyanate | NC1 | Beckman coulter |  |
|  | CD279 - PD1 | Phycoerythrin | PD1.3 | Beckman coulter |  |
|  | CD45RA | R-Phycoerythrine Texas-Red-X | 2HLDH11LDB9 (EH4) | Beckman coulter |  |
|  | CD28 | R-Phycoerythrin Cyanine 5.5 | CD28.2 | Beckman coulter |  |
|  | CD27 | R-Phycoerythrine Cyanine 7 | 1A4CD27 | Beckman coulter |  |
|  | CD8 | Allophycocyanin | SFCI21THy2D3 (T8) | Beckman coulter |  |
|  | CD4 | APC-Alexa Fluor 700 | 13B8.2 | Beckman coulter |  |
|  | CD38 | Phycoerythrin | LS198-4-3 | Beckman coulter |  |
|  | HLA-DR | R-Phycoerythrin Cyanine 7 | Immu 357 | Beckman coulter |  |
|  | CD3 | APC-Alexa Fluor 750 | UCHT1 | Beckman coulter |  |
|  | CD56 | R-Phycoerythrin Cyanine 5.5 | N901 | Beckman coulter |  |
|  | CD16 | Allophycocyanin | 3G8 | Beckman coulter |  |
|  |  |  |  |  |  |

**Supplementary Table 1.** Monoclonal antibodies clones and commercial suppliers.


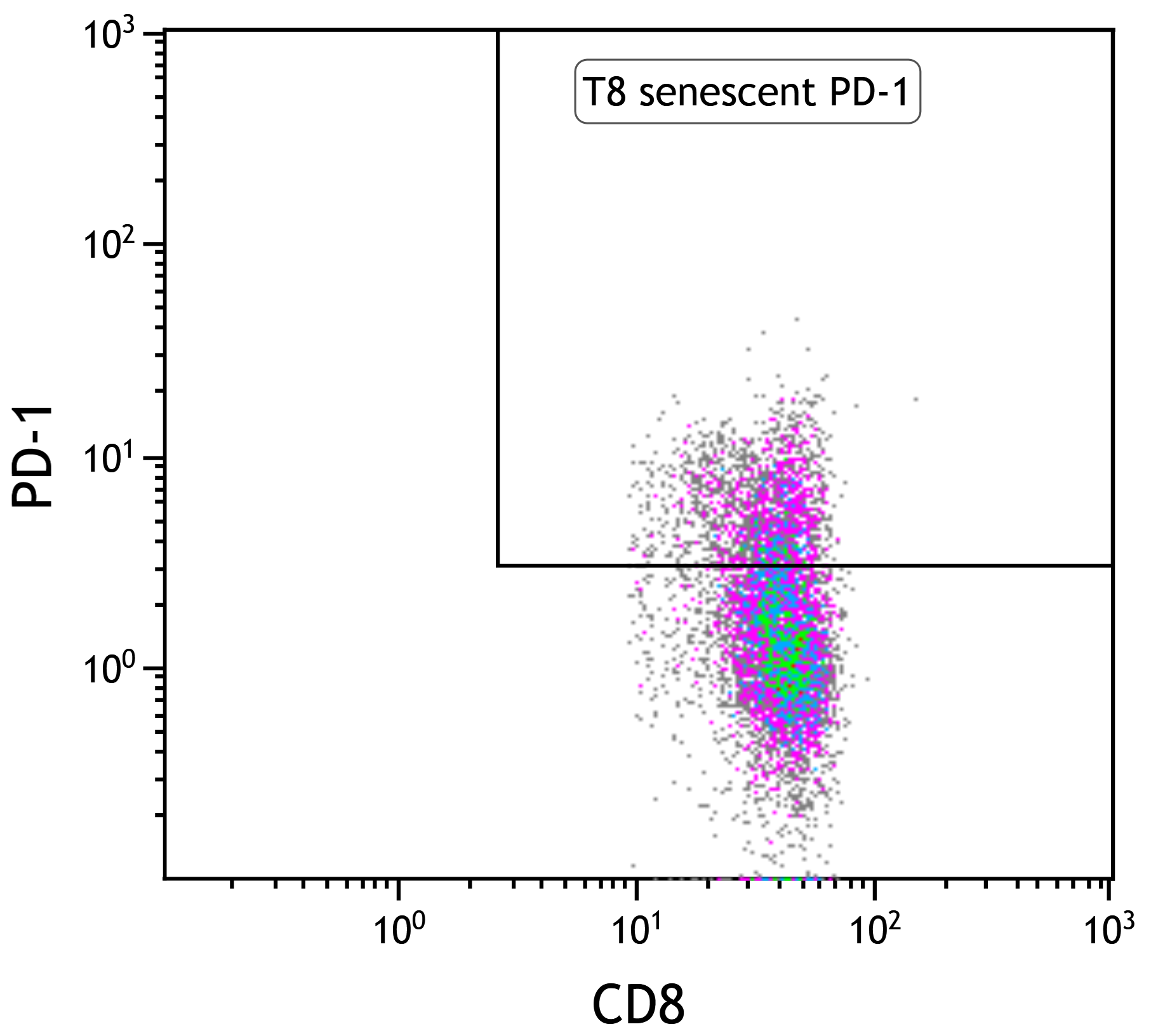

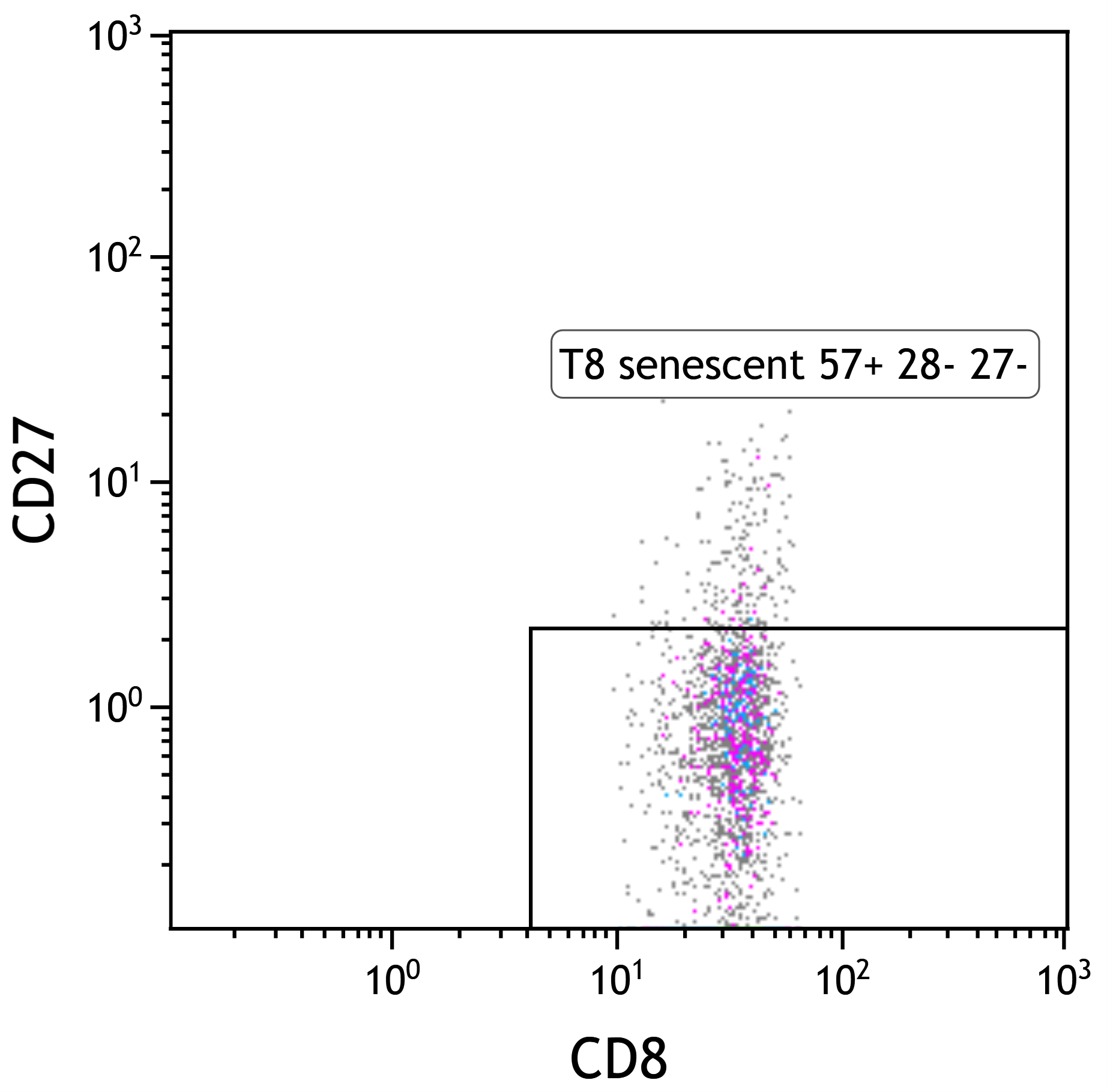

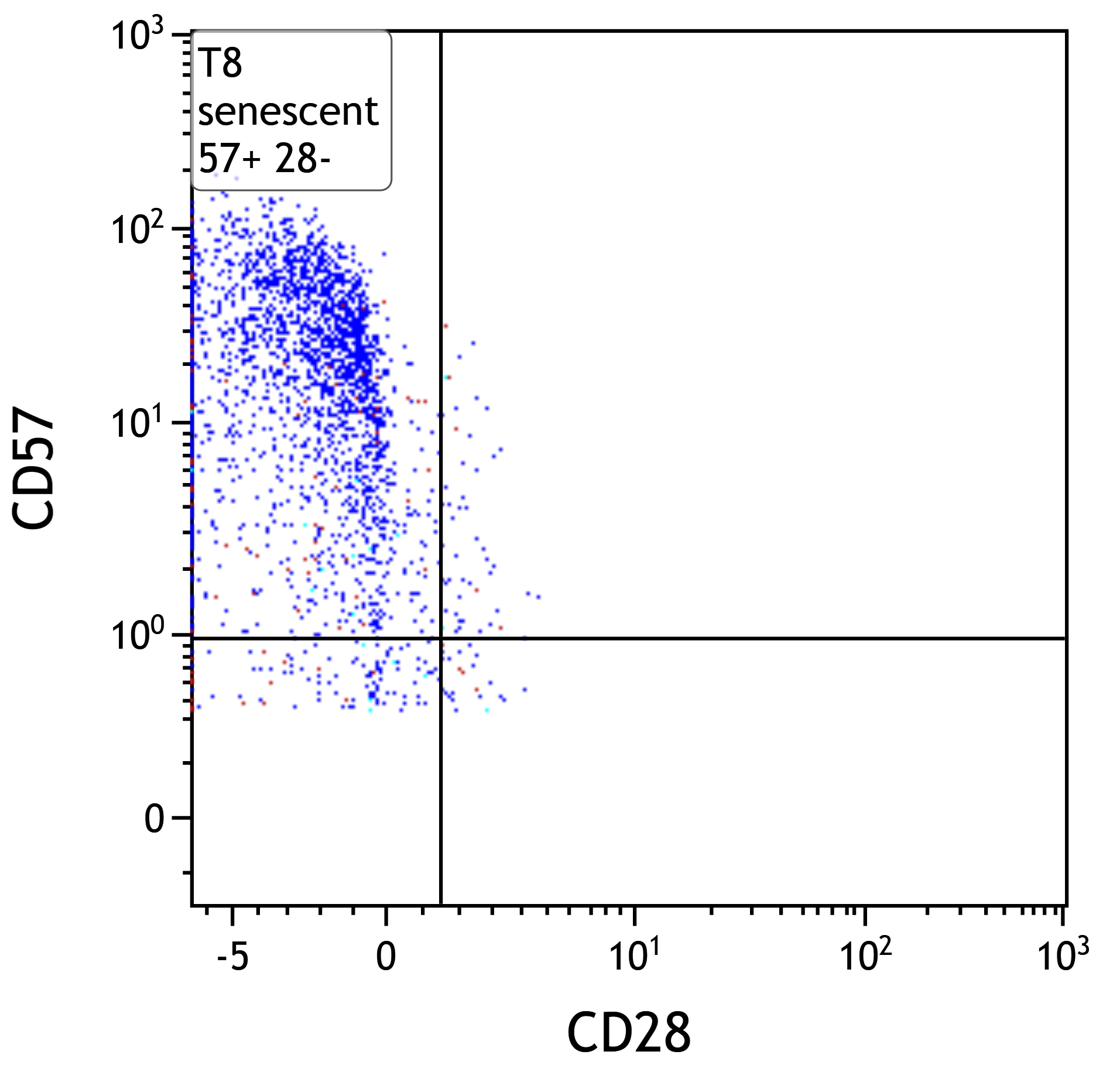

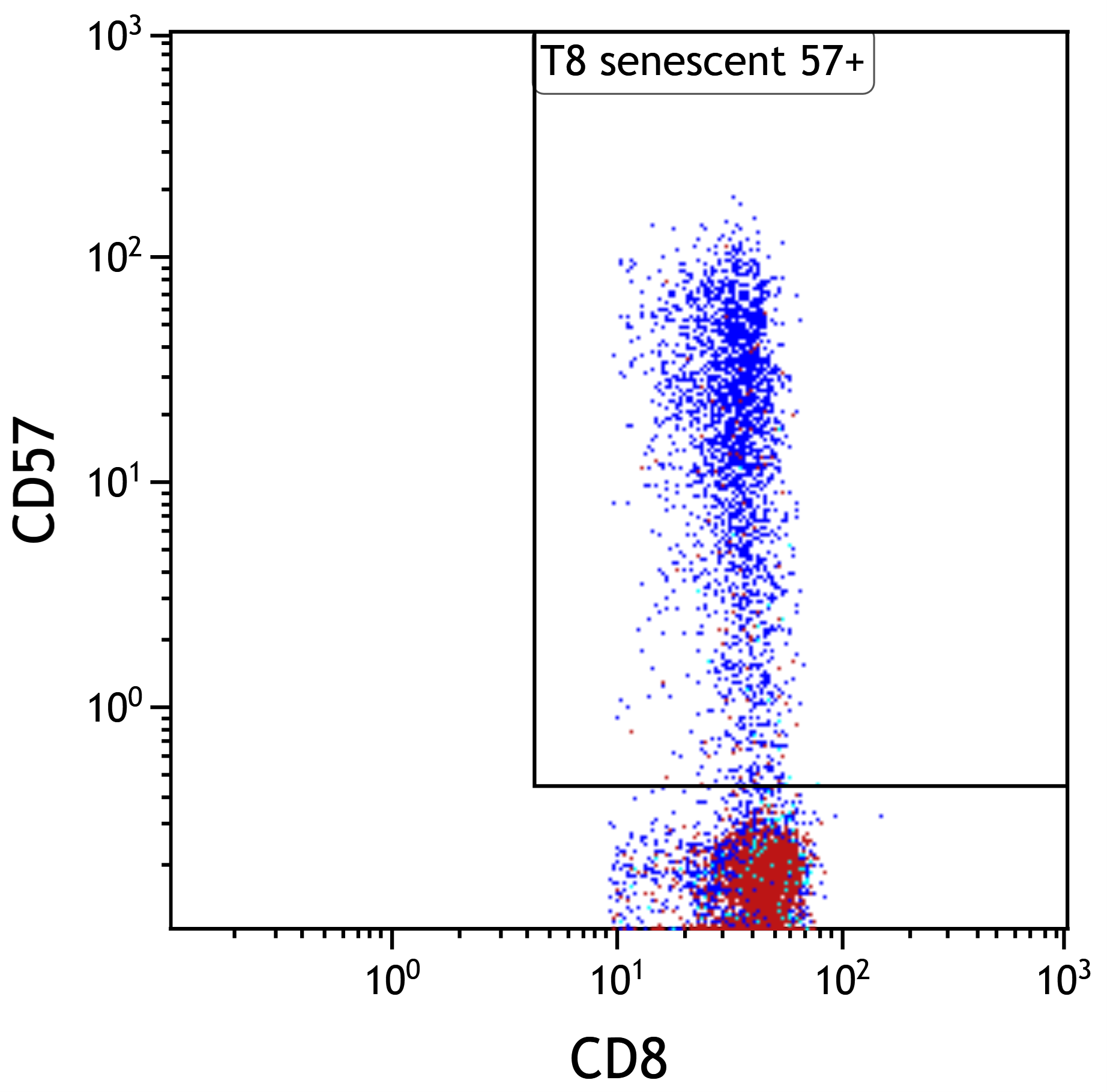

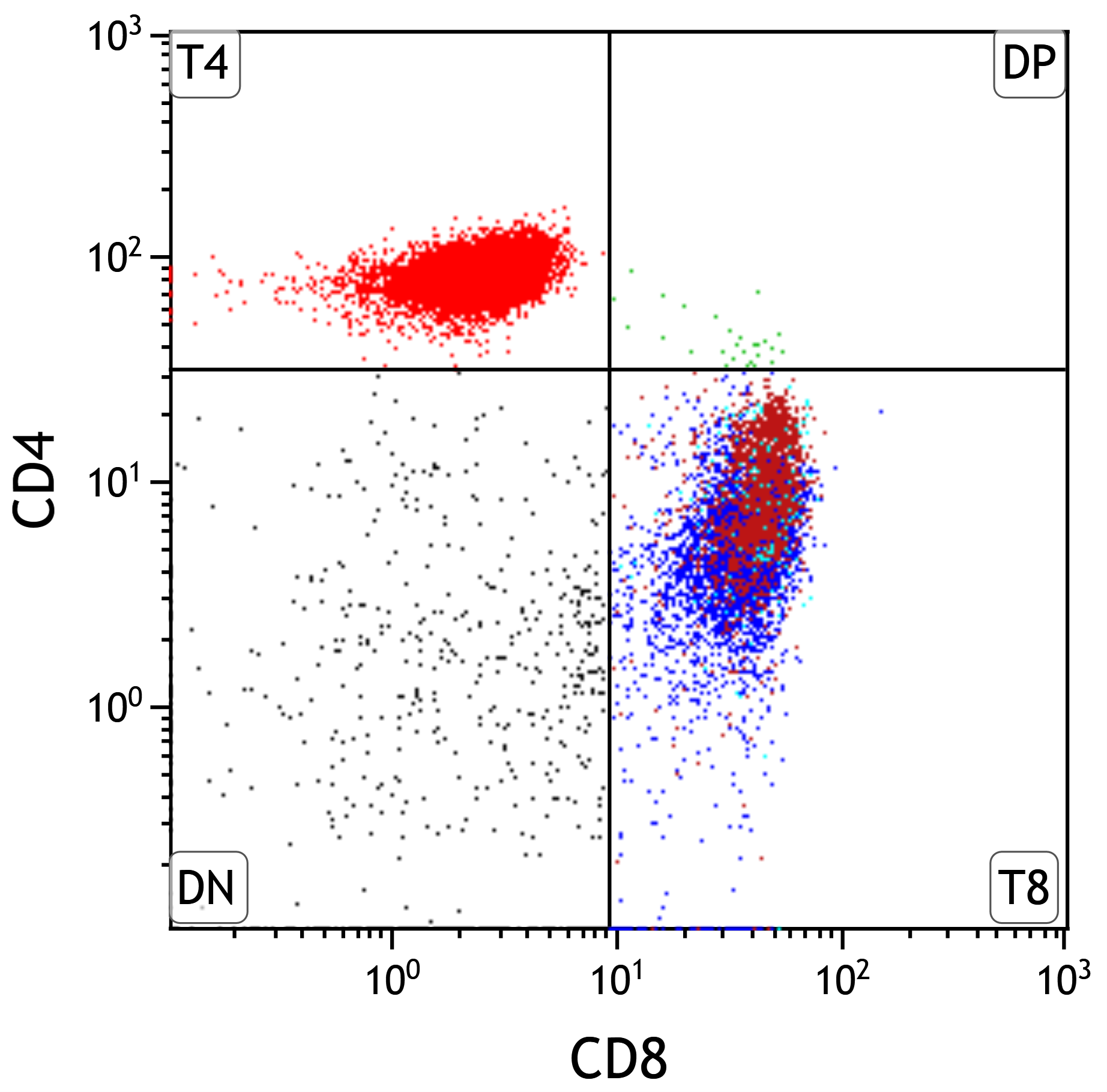

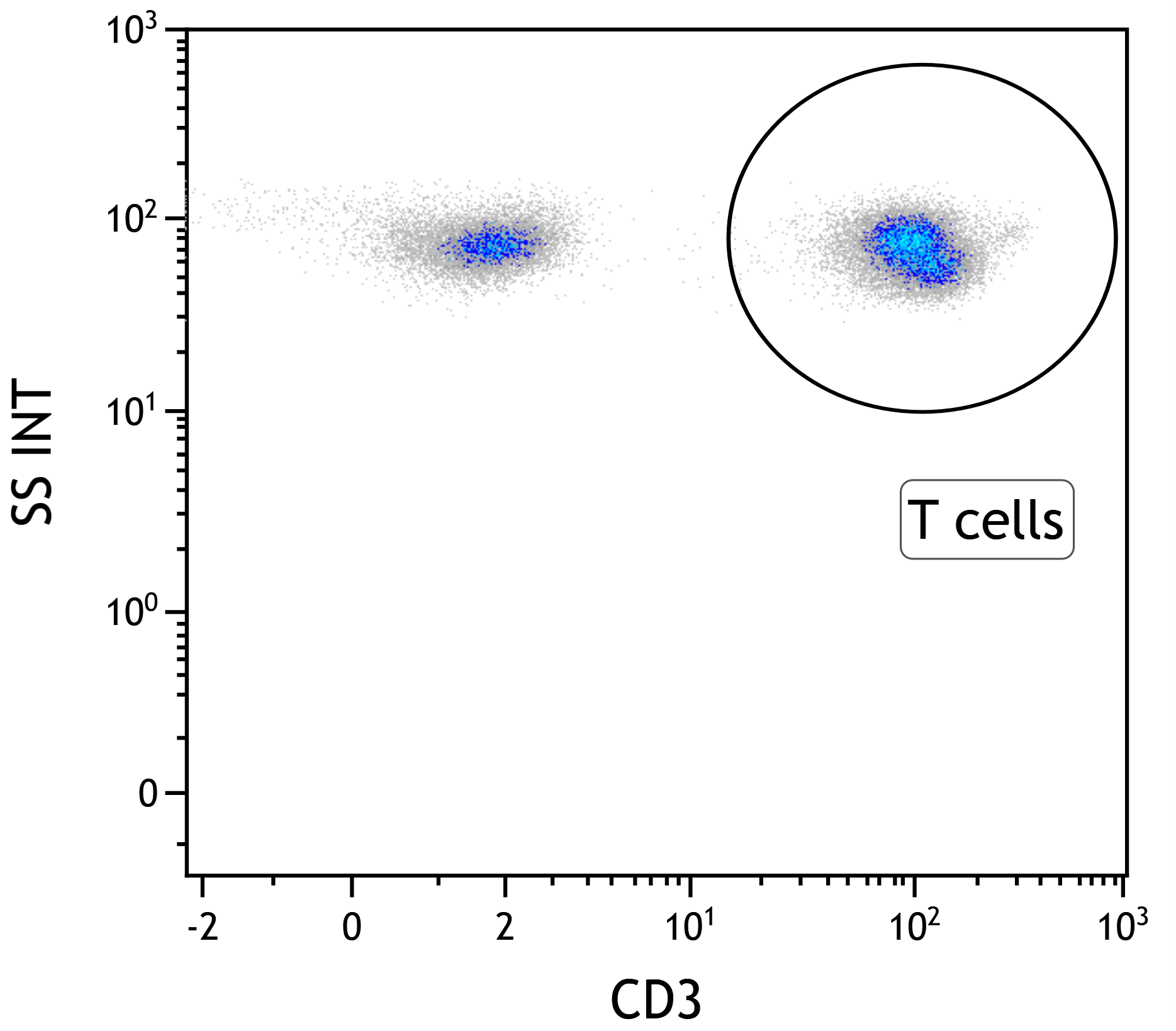

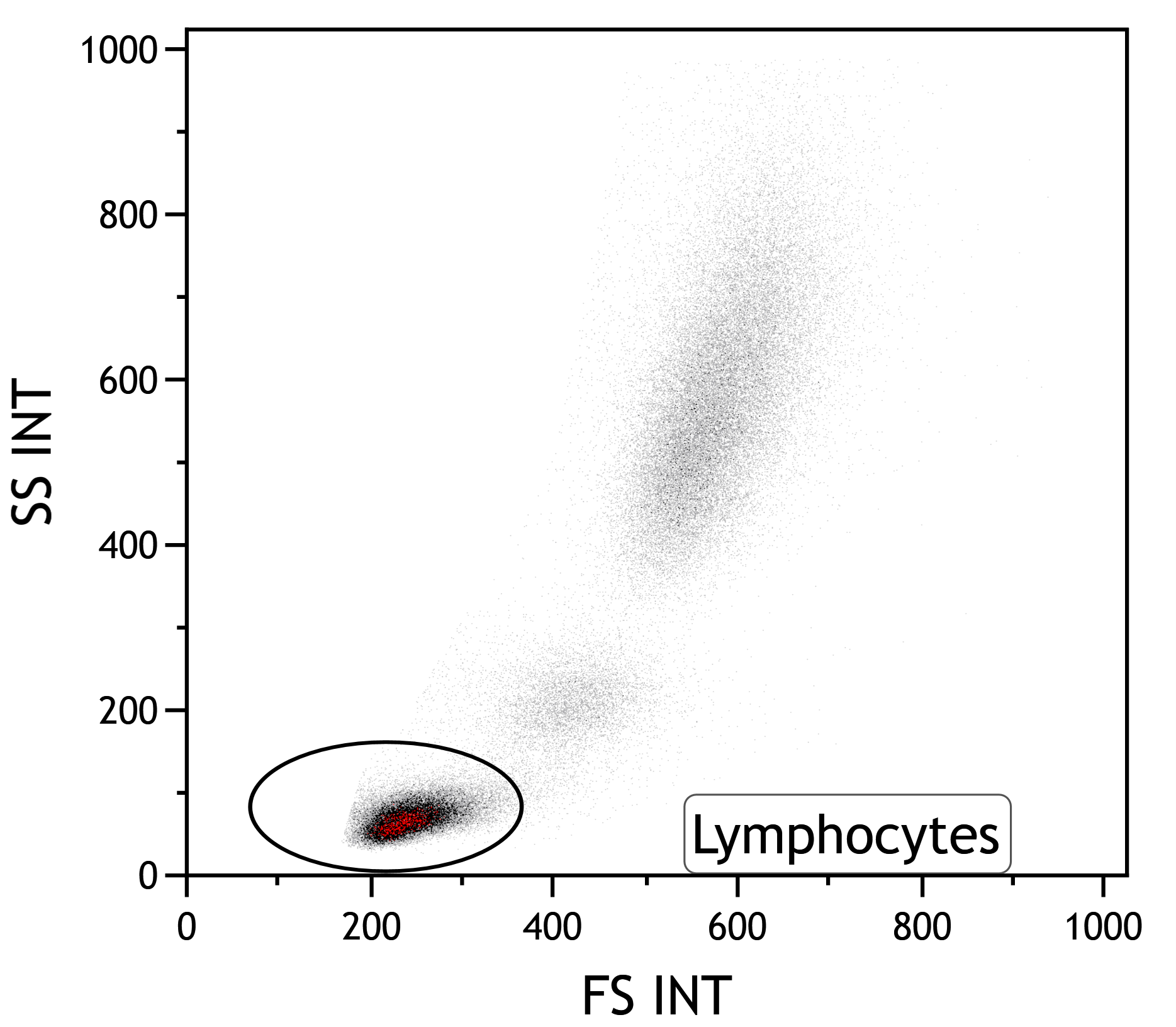


**Senescence**

**Exhaustion**

**Activation**

**Lymphocytes**


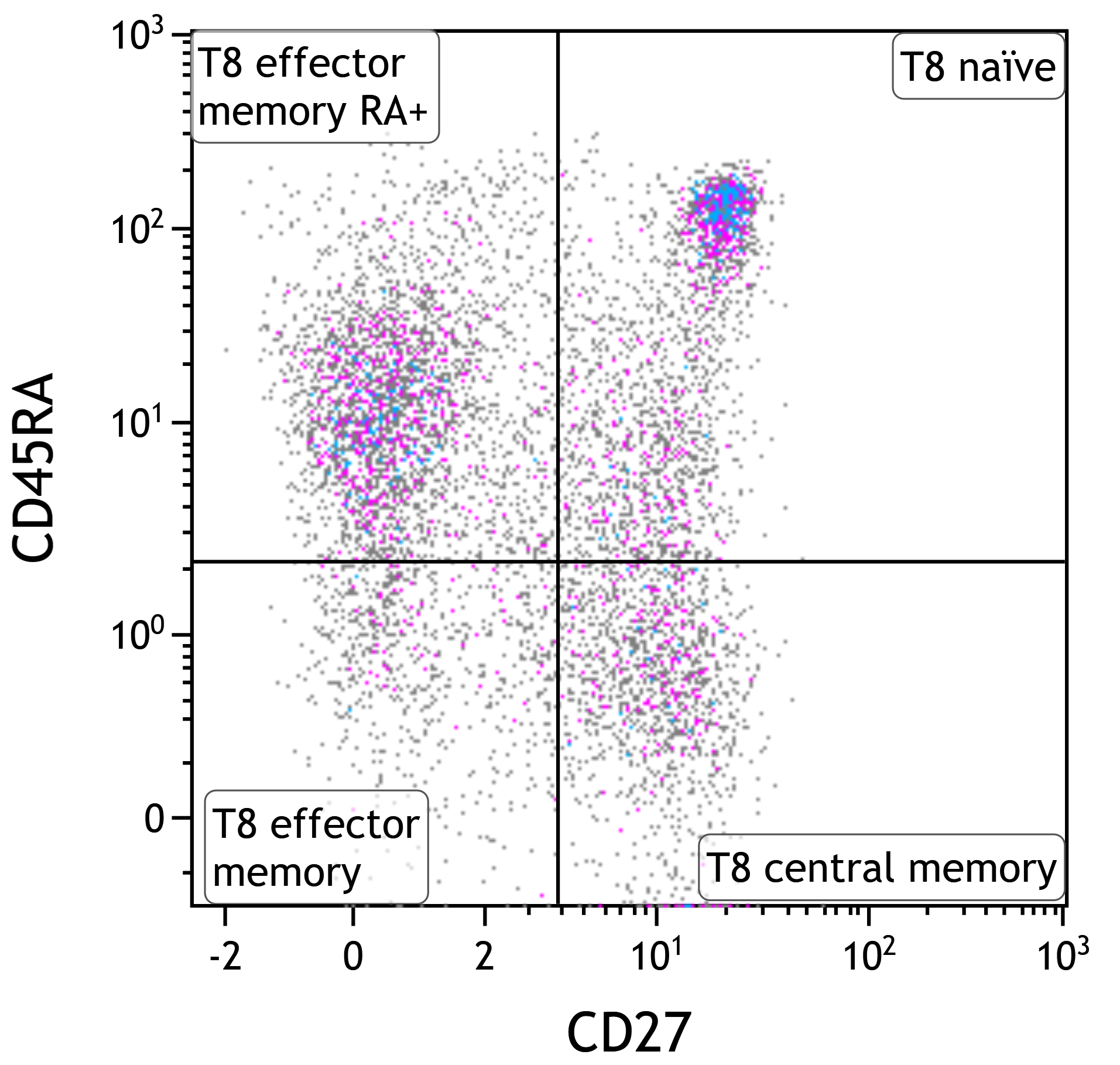

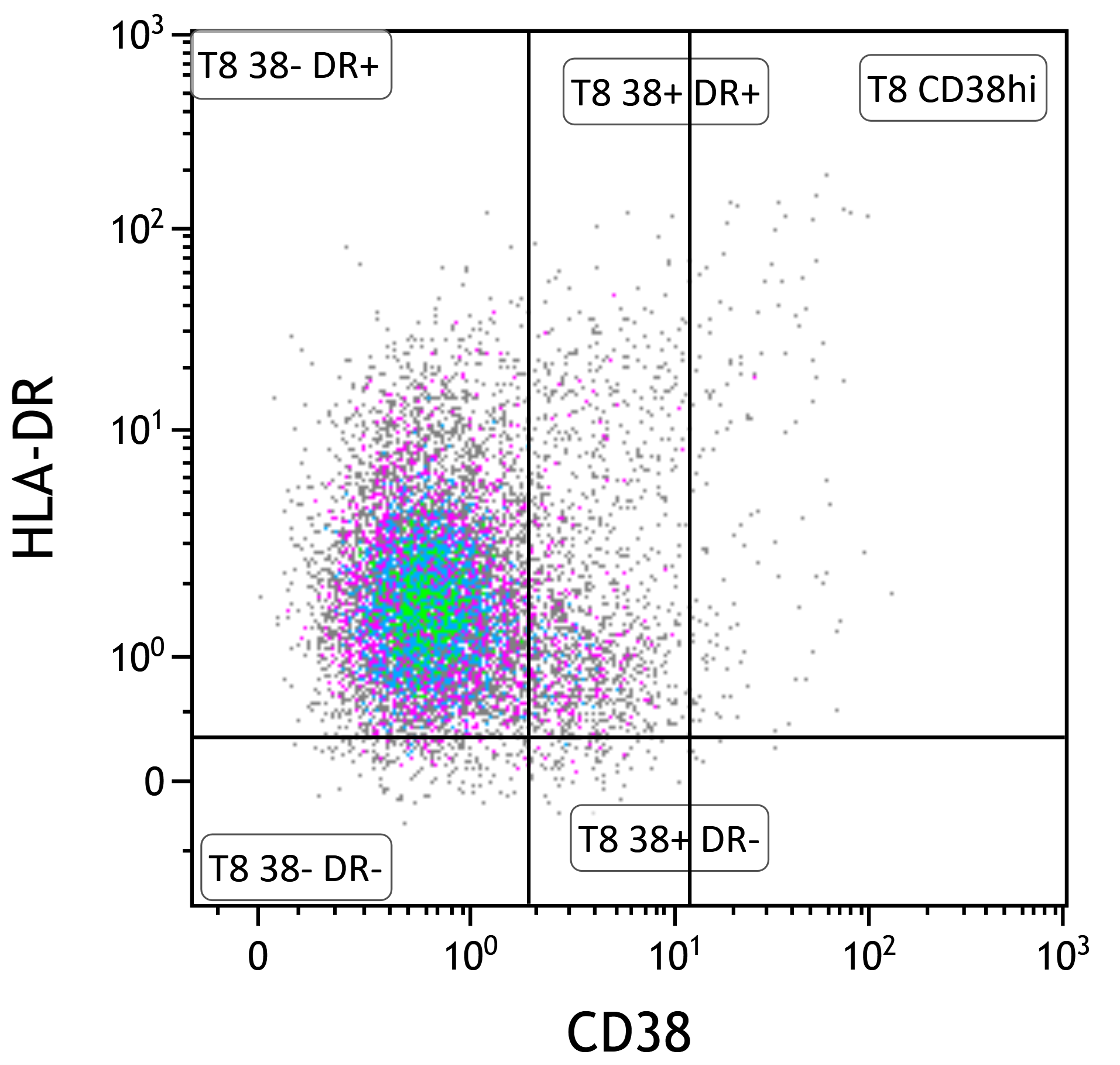


**T cells**

**CD8 T cells**

**Naïve**

**CD8 T cells**

**Effector**

**memory CD8 T cells**

**CD45RA+**

**Effector**

**memory CD8 T cells**

**CD45RA-**

**Central**

**memory CD8 T cells**

**CD38-**

**HLA-DR+**

**CD8 T cells**

**CD38+**

**HLA-DR+**

**CD8 T cells**

**CD38-**

**HLA-DR-**

**CD8 T cells**

**CD38+**

**HLA-DR-**

**CD8 T cells**

**PD-1+**

**CD8 T cells**

**CD57+**

**CD8 T cells**

**CD57+**

**CD28-**

**CD8 T cells**

**CD57+**

**CD28-CD27-**

**CD8 T cells**

**Supplementary Figure 1. Example of flow cytometry staining of CD8 T cell subpopulations.**


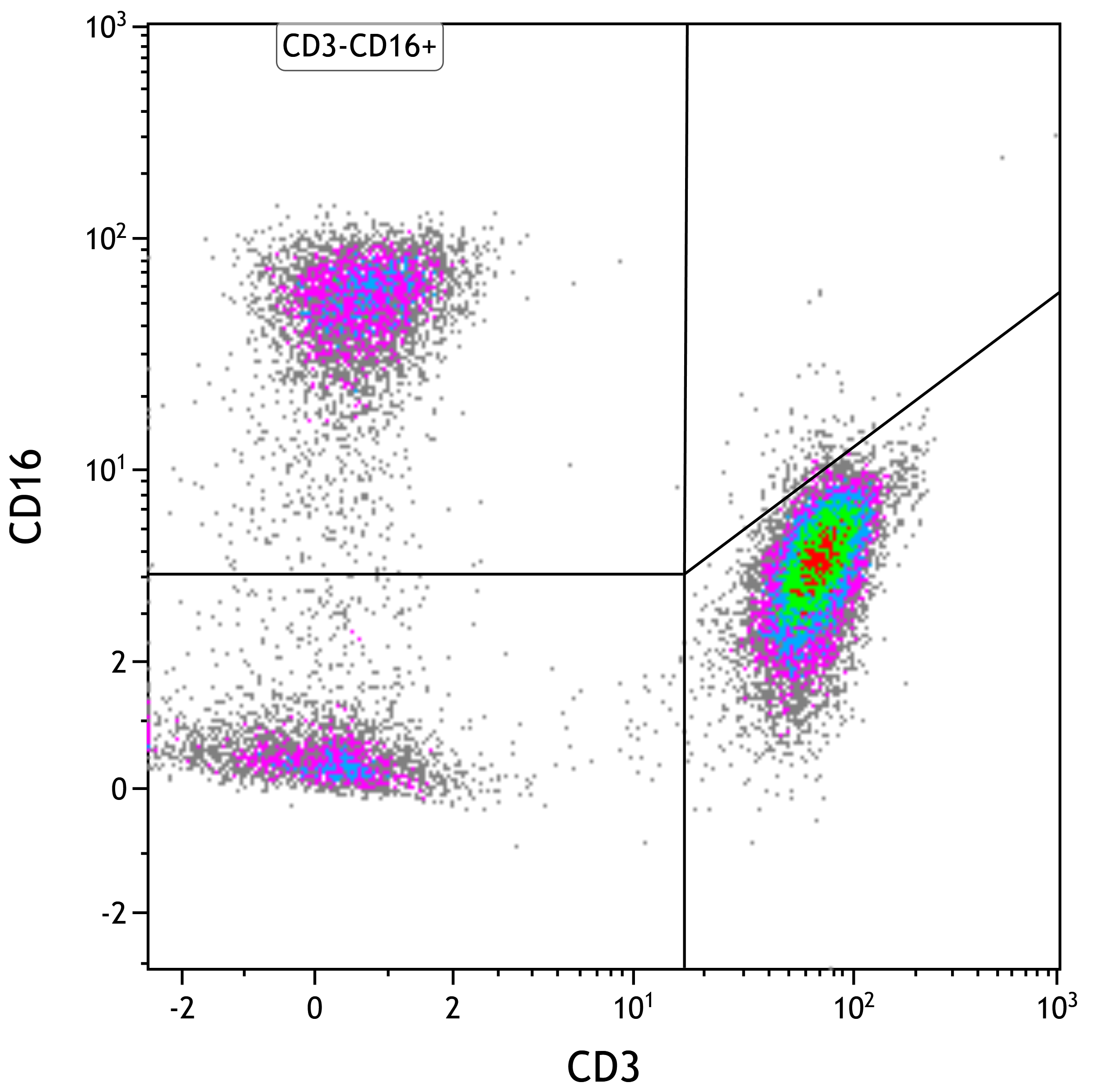

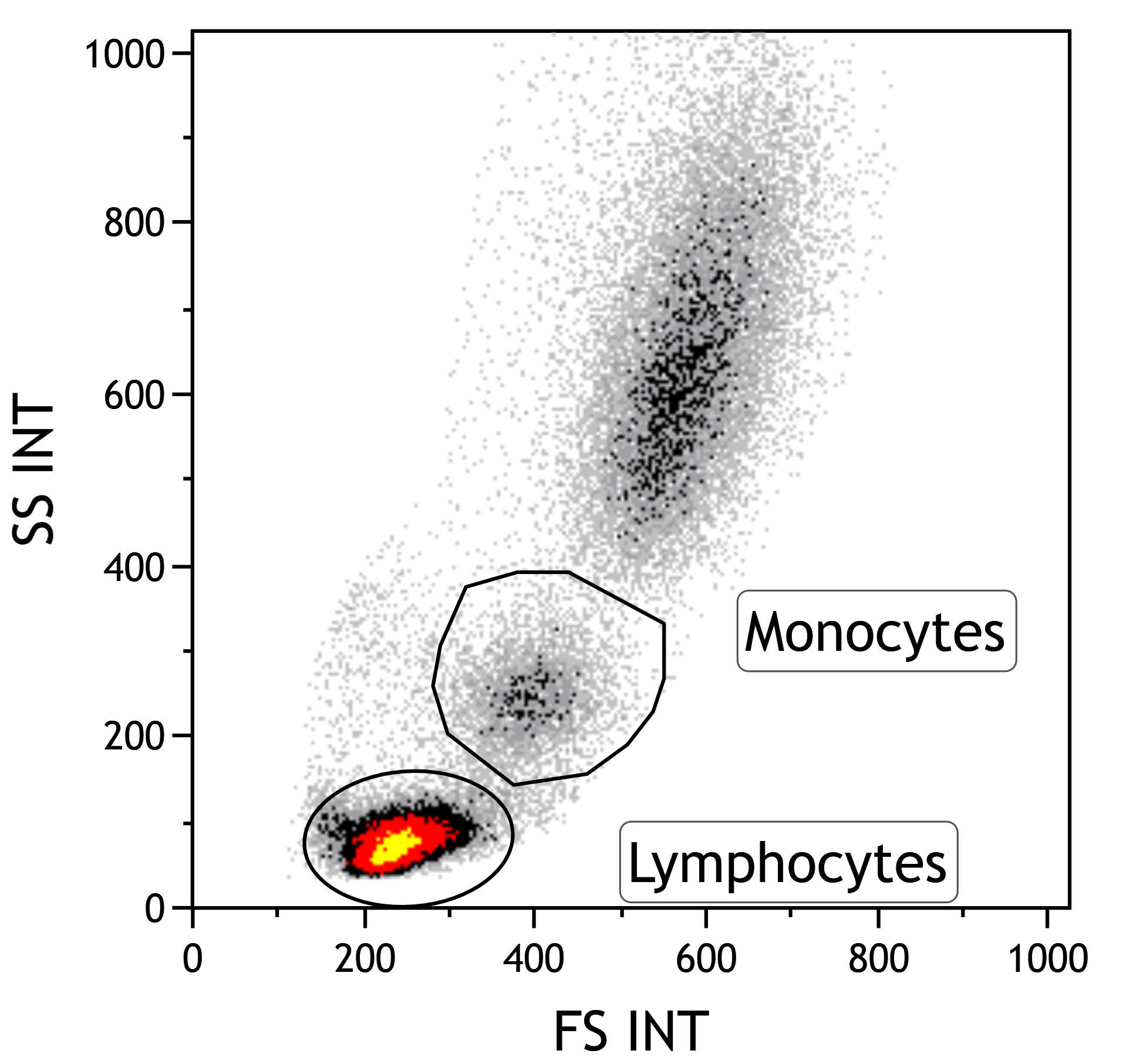

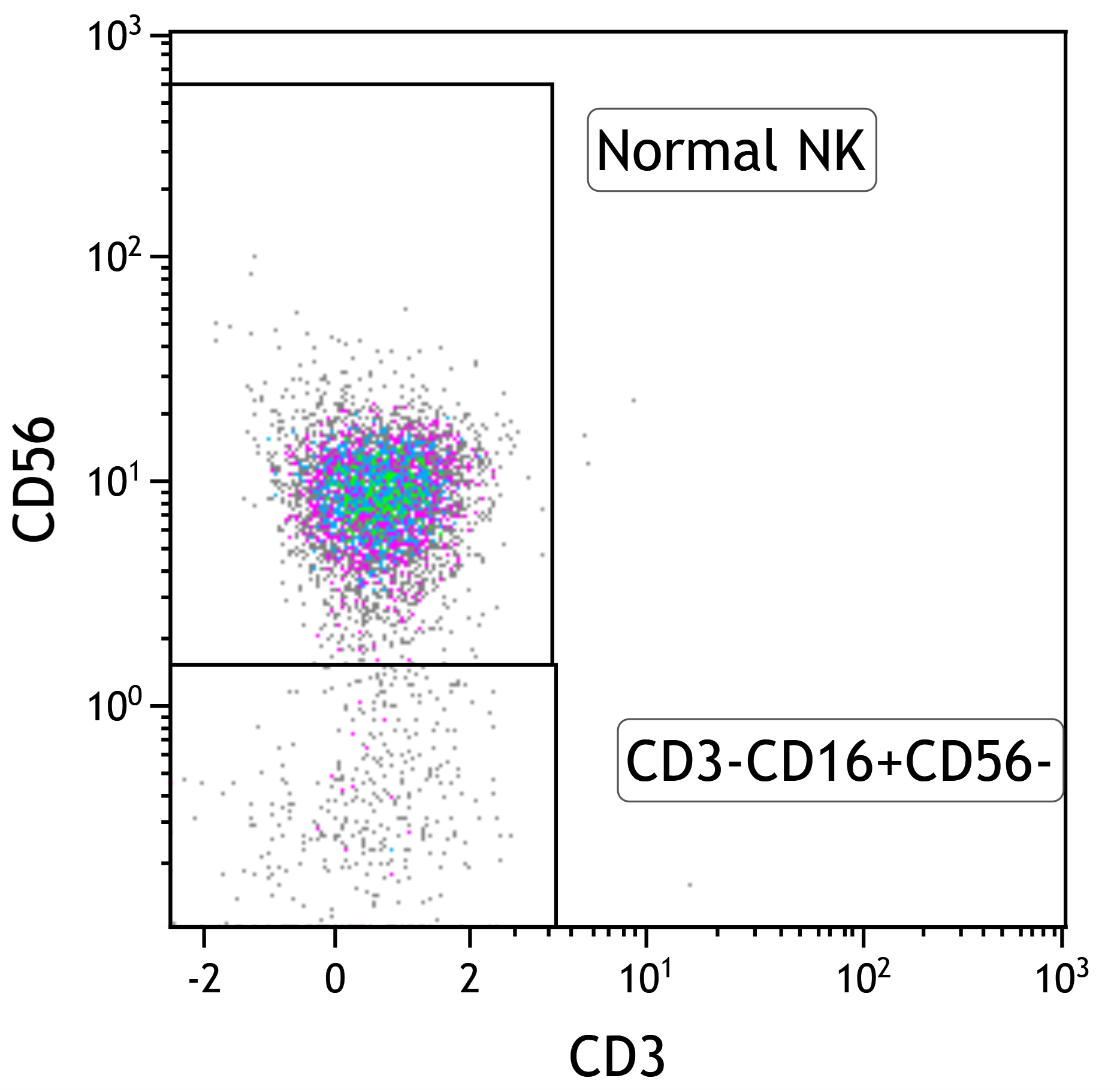

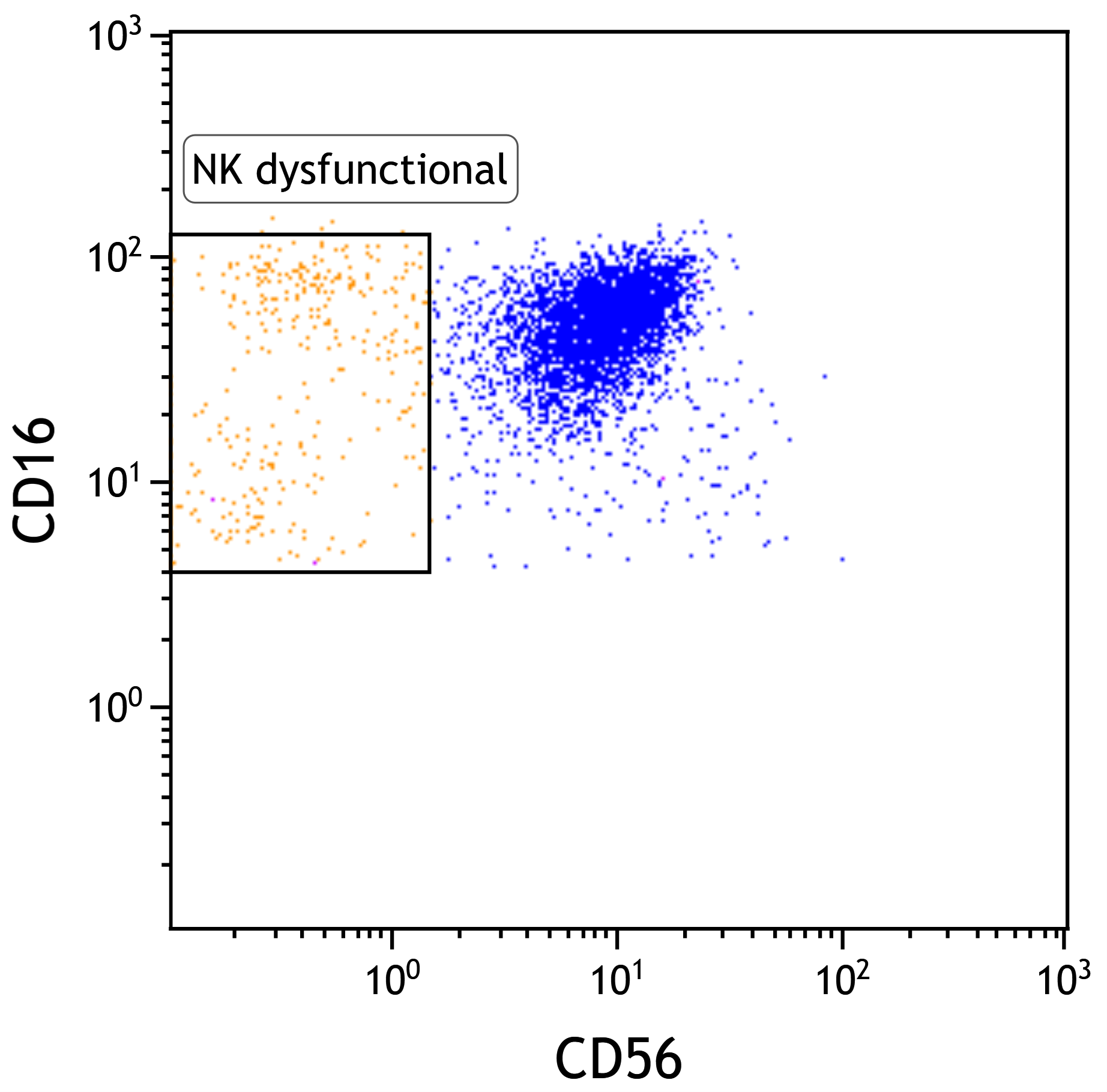

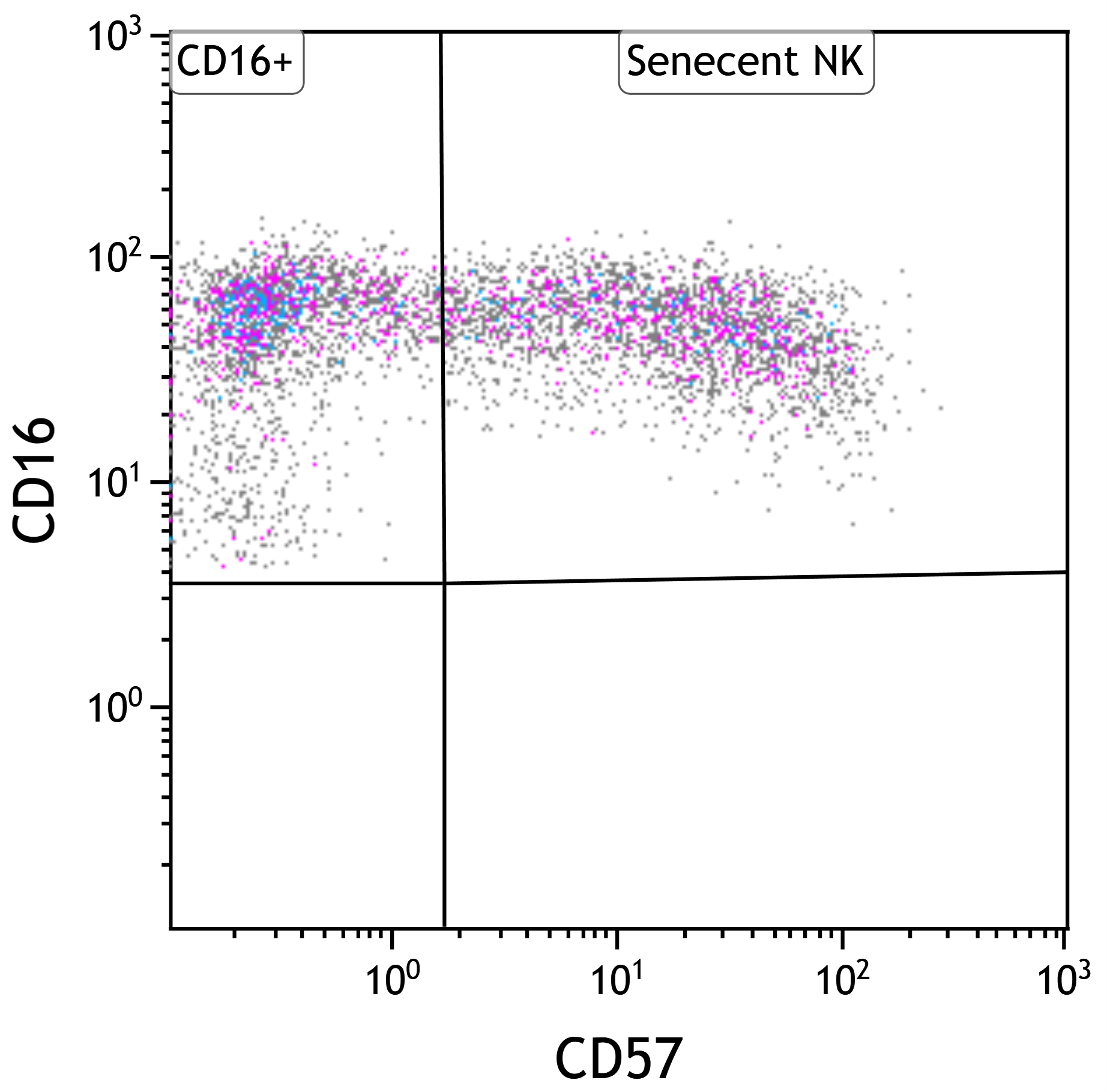


**Dysfunction**

**Senescence**


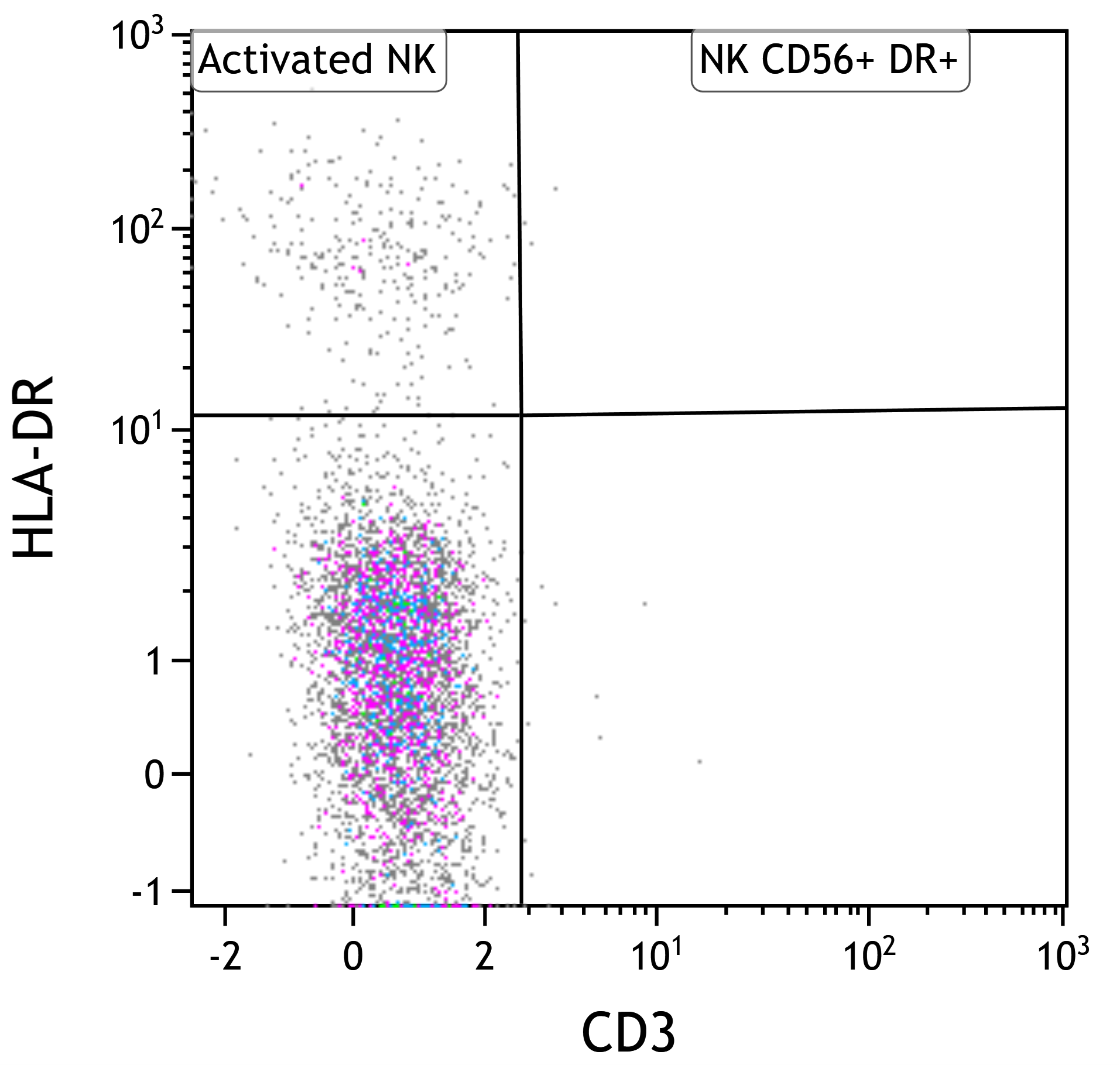


**Activation**

**Lymphocytes**

**Monocytes**

**CD56+**

**NK cells**

**CD56-**

**NK cells**

**NK cells**

**CD57+**

**NK cells**

**CD56-**

**NK cells**

**HLA-DR+**

**NK cells**

**Supplementary Figure 2. Example of flow cytometry staining of NK cell subpopulations.**
